# MicroRNA Expression Variability in Human Cervical Tissues

**DOI:** 10.1371/journal.pone.0011780

**Published:** 2010-07-26

**Authors:** Patrícia M. Pereira, João Paulo Marques, Ana R. Soares, Laura Carreto, Manuel A. S. Santos

**Affiliations:** 1 RNA Biology Laboratory, Department of Biology and CESAM, University of Aveiro, Aveiro, Portugal; 2 CIMAGO, Faculty of Medicine, University of Coimbra, Coimbra, Portugal; 3 Experimental Biology and Biomedicine PhD Program, Centre for Neurosciences, Coimbra, Portugal; Innsbruck Medical University, Austria

## Abstract

MicroRNAs (miRNAs) are short (∼22 nt) non-coding regulatory RNAs that control gene expression at the post-transcriptional level. Deregulation of miRNA expression has been discovered in a wide variety of tumours and it is now clear that they contribute to cancer development and progression. Cervical cancer is one of the most common cancers in women worldwide and there is a strong need for a non-invasive, fast and efficient method to diagnose the disease. We investigated miRNA expression profiles in cervical cancer using a microarray platform containing probes for mature miRNAs. We have evaluated miRNA expression profiles of a heterogeneous set of cervical tissues from 25 different patients. This set included 19 normal cervical tissues, 4 squamous cell carcinoma, 5 high-grade squamous intraepithelial lesion (HSIL) and 9 low-grade squamous intraepithelial lesion (LSIL) samples. We observed high variability in miRNA expression especially among normal cervical samples, which prevented us from obtaining a unique miRNA expression signature for this tumour type. However, deregulated miRNAs were identified in malignant and pre-malignant cervical tissues after tackling the high expression variability observed. We were also able to identify putative target genes of relevant candidate miRNAs. Our results show that miRNA expression shows natural variability among human samples, which complicates miRNA data profiling analysis. However, such expression noise can be filtered and does not prevent the identification of deregulated miRNAs that play a role in the malignant transformation of cervical squamous cells. Deregulated miRNAs highlight new candidate gene targets allowing for a better understanding of the molecular mechanism underlying the development of this tumour type.

## Introduction

Cervical cancer is the second most common cause of cancer-related deaths in women worldwide, incidence and mortality are, however, decreasing due to the implementation of Cervical Cancer Screening Programmes by cytological smear testing [1]. This tumour type evolves from pre-existing non-invasive pre-malignant lesions referred to as squamous intraepithelial lesions (SILs) or cervical intraepithelial lesions (CINs). These lesions are classified histologically on the basis of atypia of epithelial cells that progressively extend from the lower parabasal layers of the squamous epithelium up to the whole thickness of the epithelium, depending on the grade [2]. CINI and low-grade SIL (LSIL) correspond to mild dysplasia, CINII to moderate dysplasia and CINIII to both severe dysplasia and carcinoma *in situ*. HSIL represents the combination of CINII and CINIII. Persistent infection with high-risk types of human papillomavirus (HPV) is the causal agent for cervical neoplasia [2]. This virus contributes to neoplastic progression through the action of two viral oncoproteins E6 and E7, which interfere with critical cell cycle pathways, tumour protein p53 and retinoblastoma protein [3]. Nevertheless, evidence suggests that HPV infection alone is insufficient to induce malignant changes and other host genetic variations are important in the development of cervical cancer [2].

MicroRNAs (miRNAs) are a class of evolutionary conserved non-coding RNAs that regulate stability and translation efficiency of target mRNAs [4] and have a direct impact on cancer development [5]. These RNAs are 19 to 25 nt long and are cleaved from 70 to 100 nt hairpin pre-miRNA precursors. The precursors are cleaved by cytoplasmic RNase III Dicer into ∼22 nt miRNA duplex: one strand (miRNA*) of the short-lived duplex is degraded, whereas the other strand, which serves as mature miRNA, is incorporated into the RNA-induced silencing complex (RISC) and drives the selection of target miRNAs containing antisense sequences [4]. They are likely to control expression of thousands of genes, suggesting that they play fundamental global roles in human biology, including development, differentiation, apoptosis, metabolism, viral infection and cancer [4].

Comparison between human cancer and their normal tissues counterparts have revealed distinct miRNA expression profiles. Several studies have shown that miRNAs are aberrantly expressed or mutated in tumours and recent data strongly suggests that miRNA profiling is more robust than mRNA profiling in tumour classification [6], [7]. Moreover, a growing number of miRNAs have been implicated in promoting or suppressing tumorigenesis in a variety of tissues [8]–[10], suggesting that they may play a role as a novel class of oncogenes or tumour suppressor genes. This is supported by the observation that the 13q14 deletion, which is present in more than half of all chronic lymphocytic leukemias (CLL) results in loss of *miR-15a* and *miR-16-1* genes [11]. And the mir-17/92 cluster cooperates with the oncogene *Myc* during tumour development in a mouse model [12], while miR-372 and miR-373 cooperate with the *RAS* oncogene in an *in vitro* assay [13]. Finally, more than 50% of miRNA genes are located in chromosome domains that are genetically altered in human cancer [14].

The role of miRNAs in cervical cancer is still poorly understood, however various studies have already been carried out. Lui *et al.* have characterized the profiles of miRNAs and other small RNA segments in six human cervical cell lines and five normal cervical samples using a direct sequencing method [15]. They found reduced expression of miR-143 and increased expression of miR-21 in 29 matched pairs of human cervical cancer and normal cervical specimens [15]. Another study showed that miRNA profiles in cervical squamous cell carcinoma depend on Drosha, which is an RNase III enzyme involved in the miRNA biogenesis pathway [16]. Martinez and co-workers have demonstrated that HPV alter the expression of miRNAs in cervical carcinoma cell lines [17]. In a fourth study, 10 early stage invasive squamous cell carcinomas (ISSC) and 10 normal cervical squamous epithelial biopsies were profiled for miRNA misexpression using TaqMan real-time quantitative PCR [18]. This study identified 68 up-regulated and 2 down-regulated miRNAs between the ISCCs and normal epithelial tissues, with miR-199s, miR-9, miR-199a*, miR-199a, miR-199b, miR-145, miR-133a, miR-133b, miR-214 and miR-127 being among the miRNAs most overexpressed. By contrast, only two of the miRNAs, miR-149 and miR-203 showed significant down-regulation [18]. A study analyzing eight cervical cancer cell lines, two HPV16+ W12 subclones [19] and five age-matched normal cervix and cervical cancer tissues was also reported [20]. The authors showed that miR-126, miR-143 and miR-145 were down-regulated and miR-15b, miR-16, mi-146 and miR-155 were up-regulated. Their data also indicated that decreased miR-143 and miR-145 expression and increased miR-146a expression are relevant for cervical carcinogenesis. Finally, Hu and co-workers have recently identified miR-200a and miR-9 as predictors of patient survival in cervical carcinoma [21]. These studies were unable to clarify the role of miRNAs in cervical cancer due to inconsistency in miRNA expression between them, which may be due to differences in the high-throughput platforms and methods used in different laboratories or due to differences among the cancer population. Also, a full characterization of the complex relationship between miRNAs and their target mRNAs in cervical malignant transformation has not yet been carried out.

We present the results of miRNA expression profiling in cervical squamous cell carcinomas (SCC), low and high-grade intraepithelial cervical lesions and normal cervical epithelial tissues. As in other studies, we have observed high expression variability between samples, especially among normal cervical samples, which did not allow us to obtain a unique miRNA expression signature for this tumour type. We demonstrate using Taqman miRNA real-time PCR quantification method that such variability is biological rather than technical. We have tackled such biological variability by pooling the RNAs from the normal samples, which averaged miRNAs levels in the controls, and we demonstrate that this methodology is sufficiently robust to identify miRNAs that were deregulated between malignant, pre-malignant and normal cervical tissues, which may be involved in cervical carcinogenesis. We also identify possible gene targets of relevant candidate miRNAs.

## Results

### High variability of miRNA expression in cervical tissues

**Figure 1:**
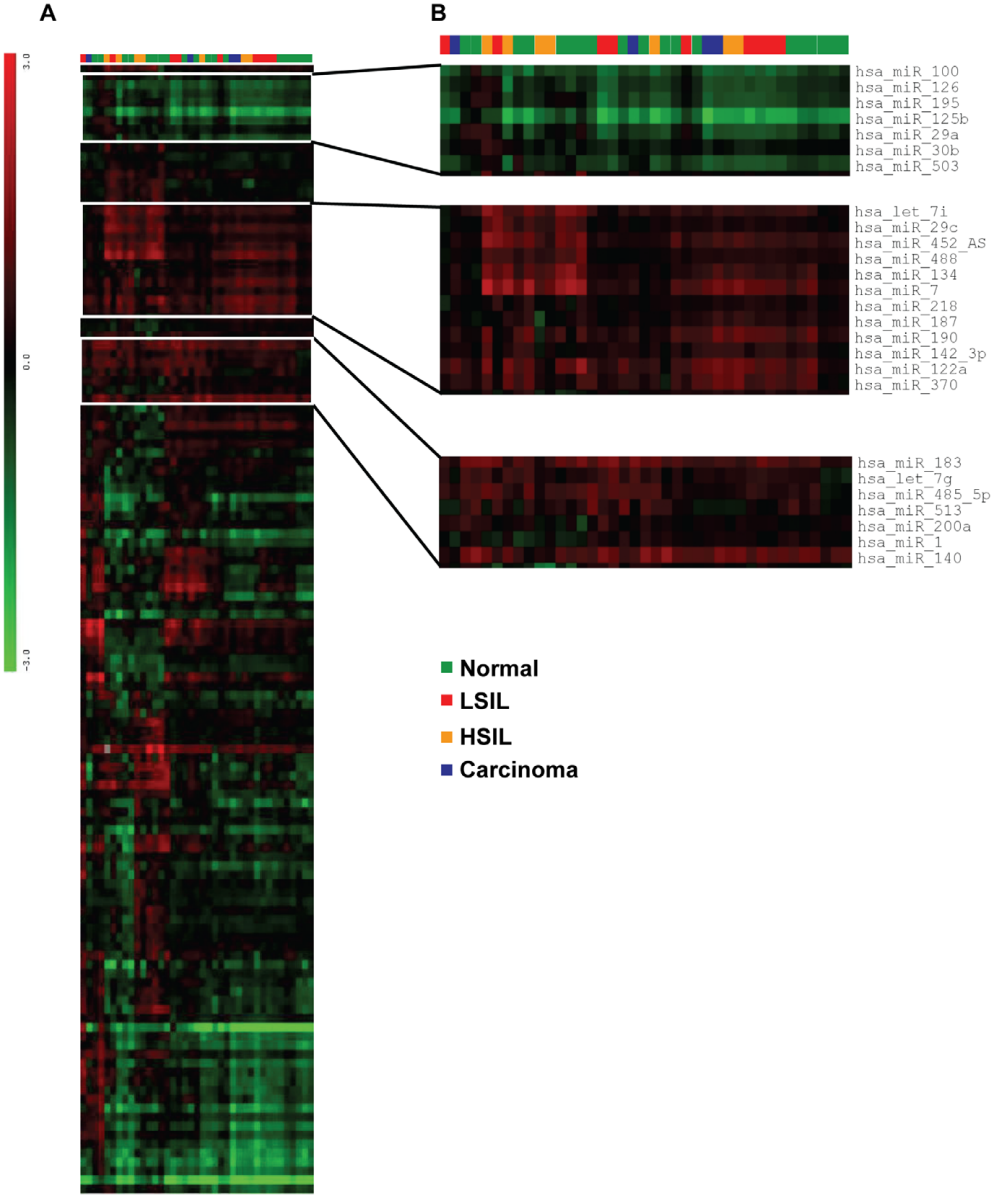
Cluster analysis of cervical samples. **A**) Tree generated by unsupervised hierarchical clustering (Pearson correlation, average linkage) of 25 cervical samples (columns) and 126 miRNAs (rows). **B**) Zoom-in of some of the miRNAs showing that samples were not grouped according to their histological classification. The profiles identified are relative to commercial RNA from normal cervix. Blue, carcinoma; Yellow, High-grade Intraepithelial Lesion (HSIL); Red, Low-grade Intraepithelial Lesion (LSIL); Green, Adjacent normal tissue. Colours in the heatmap indicate: green, lower expression compared to the mean (zero); black, expression equal to the mean; gray, absent data points and red, higher expression compared to the mean.

We used a miRNA microarray spotted in house to analyze miRNA expression in four cervical squamous cell carcinomas (SCC), five high-grade intraepithelial lesions, nine low-grade intraepithelial lesions and 19 normal cervical tissues; a total of 25 biologically independent samples (Table 1). These tissues were initially snap-frozen in liquid nitrogen and then stored at −80°C until used. A pool of four commercial RNAs from normal cervix (Ambion) was used as common reference. Following RNA hybridization and array analysis, samples were clustered according to their miRNA expression profile using the hierarchical clustering algorithm of the MeV 4.0.01 software package (Figure 1A) but, surprisingly, the generated tree showed no clear distinction of samples regarding their histological classification (Figure 1B, coloured top bar). Also, we could not identify miRNAs with significant down- or up-regulation in pre- and malignant samples versus normal cervix (Figure 1B, heat map clusters zoomed-in). We were not able to find differentially expressed miRNAs between all samples, according to their histological classification. The overall heat map did not show clusters with different miRNA expression pattern between normal, pre- and malignant samples (Figure 1A).

**Table 1:** Clinicopathologic background of 25 patients used in this study.

		Descriptive characteristics of patients			
Patient #	Age	Lesion type	FIGO stage	Human Papillomavirus	Microarray
1	32	LSIL/CINI		+	L2, N9
2	49	HSIL/CIN II		−	H3, N8
3	44	LSIL/CIN I		+	L1, N16
4	51	LSIL/CINI		+	L10
5	44	LSIL/CIN I		+	L5, N4
6	30	LSIL/CIN I			N7
7	40	HSIL/CIN II		+	H1
8	25	LSIL/CIN I		+	L6, N1
9	46	LSIL			N6
10	33	LSIL/CIN I		−	L11, N17
11	35	LSIL/CIN I			N5
12	25	HSIL/CIN III		+	H4, N2
13	50	SCC	IA1	+	C3, N3
14	28	HSIL/CIN III		+	H5
15	21	HSIL/CIN III		+	H6, N12
16	39	HSIL/CINIII/CIS		+	H7
17	33	LSIL/CIN I		+	L7, N13
18	27	HSIL/CIN III		−	H2, N10
19	44	SCC	0	+	C4, N11
20	27	LSIL/CIN I		+	L8, N14
21	30	LSIL/CIN I		+	L9, N15
22	47	SCC	IB1	+	C5
23	29	No lesion			N18
24	28	No lesion			N19
25	30	SCC	IIB	+	C6

**Figure 2:**
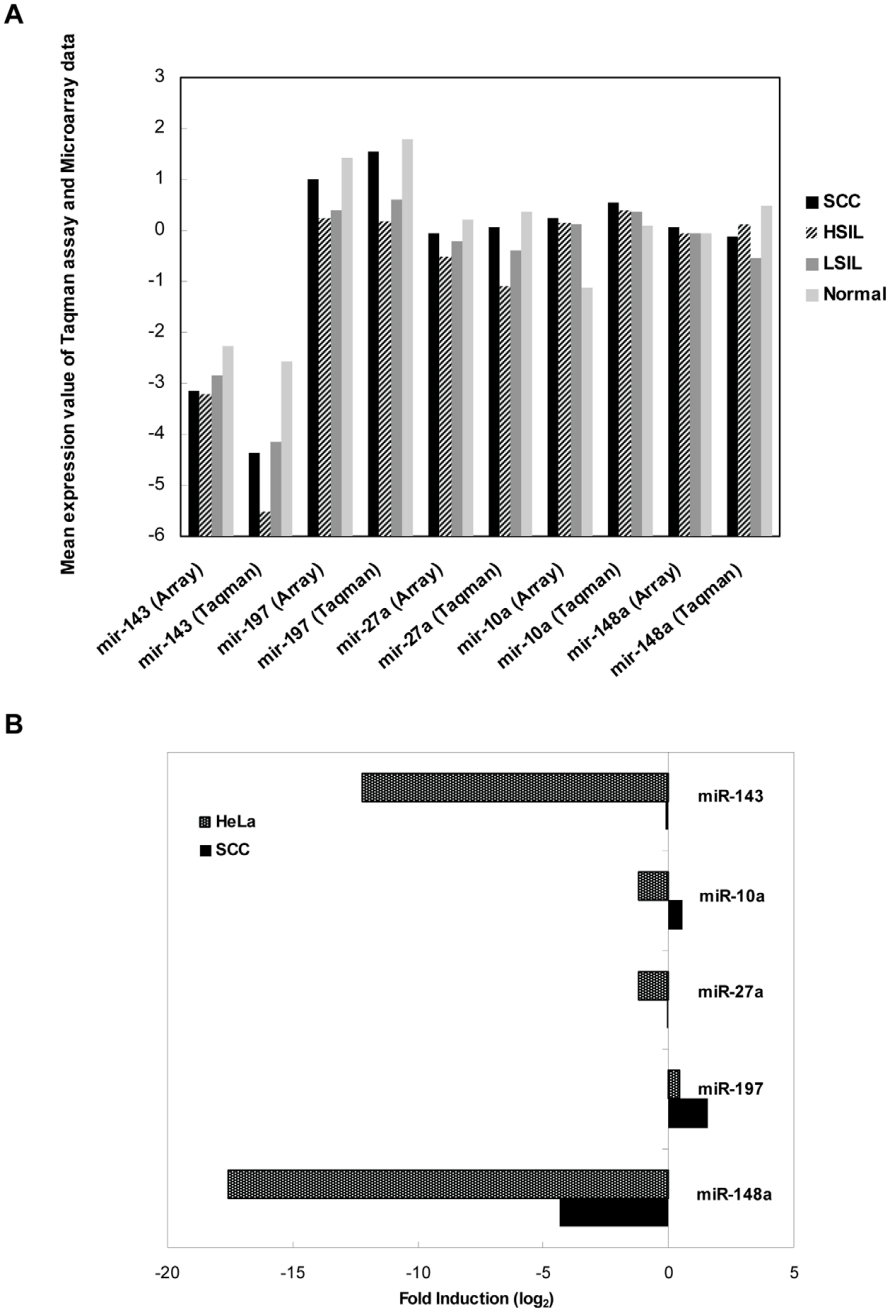
Expression of 5 miRNAs quantified by Taqman quantitative real-time PCR assay. **A**) Comparison of miRNA expression levels obtained by spotted arrays and Taqman qRT-PCR analysis of 3 cervical cancers (black), 3 High-grade Intraepithelial Lesions (diagonal), 3 Low-grade Intraepithelial lesions (dark grey) and 1 normal pooled sample (light grey) compared with normal cervix reference. **B**) miRNA expression measurement in HeLa cells and cervical cancers by Taqman qRT-PCR compared with normal cervix reference. The abundance of each miRNA in a total RNA sample was normalized to the level of the RNU6 in the same sample.

In order to validate or reject the microarray data and to ensure that the variability observed among samples was not technical we carried out a Taqman miRNA quantitative real-time PCR analysis of five miRNAs of three cervical carcinoma, six atypical dysplasia (CINIII, n = 3; CINI, n = 3) and 1 normal pooled samples. Each miRNA was quantified in each sample and its expression level was normalized to that of the RNU6 and to commercial normal cervix RNA, which was used as reference. The miRNA expression patterns from normal to cervical cancer were consistent between the microarray and Taqman measurements (Figure 2A). This confirmed that the variability found in the miRNA profiles was biological rather than technical. In addition, we also performed Taqman miRNA assay in a HeLa cell line (Invitrogen) and we compared it with cervical carcinoma samples data (Figure 2B).

**Figure 3:**
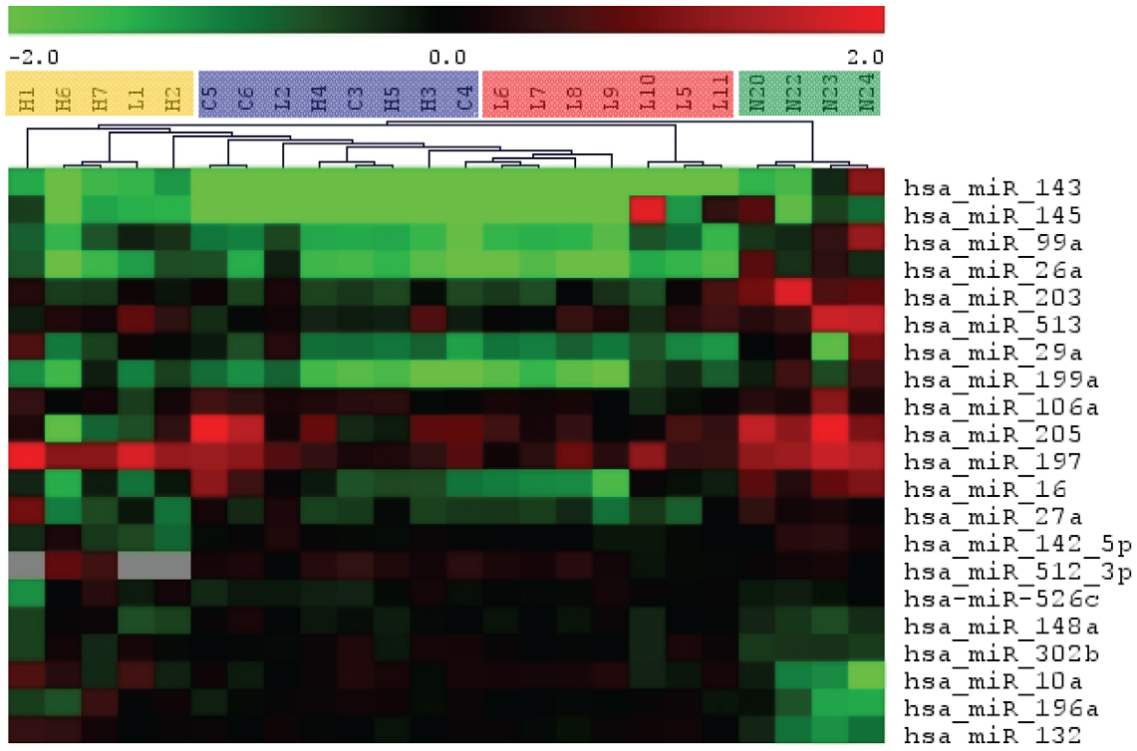
Unsupervised hierarchical clustering of 21 miRNAs whose expression was deregulated. (Pearson correlation, average linkage). The 24 cervical samples are indicated in the columns and 21 miRNAs are indicated in the rows. C, carcinoma; H, High-grade Intraepithelial Lesion; L, Low-grade Intraepithelial Lesion; N, Adjacent normal tissue pool. The colour scale at the top of the panel represents the degree of expression. Green indicates lower expression than the mean (zero), black indicates expression equal to the mean, gray indicates absent data points, and red indicates higher expression compared to the mean. Green represents the group of normal samples, red represents the low-grade intraepithelial lesion group, blue represents the high-grade intraepithelial lesion/cervical carcinoma group and yellow represents the high-grade intraepithelial lesion group (with exception of L1).

In an attempt to overcome or minimize such variability in the normal samples we have prepared a RNA pool containing equal amounts of total RNAs extracted from the normal cervical biopsies and we hybridized it on the miRNA arrays as described for the other samples, and performed Taqman miRNA assay. Thus, we were able to reduce sample variation due to the presence/absence of HPV and patient's age, this method allowed us to dilute inter-individual variability. To assess whether this strategy was effective we have performed unsupervised hierarchical clustering of the miRNAs that were differentially expressed between samples (21 miRNAs). Three groups representing: normal samples (N); low-grade intraepithelial lesion (L) (exceptions were samples L1 and L2) and high-grade intraepithelial lesion (H)/cervical carcinoma (C), were then visible in the cluster analysis (Figure 3).

### Differentially expressed miRNAs in cervical cancer

**Figure 4:**
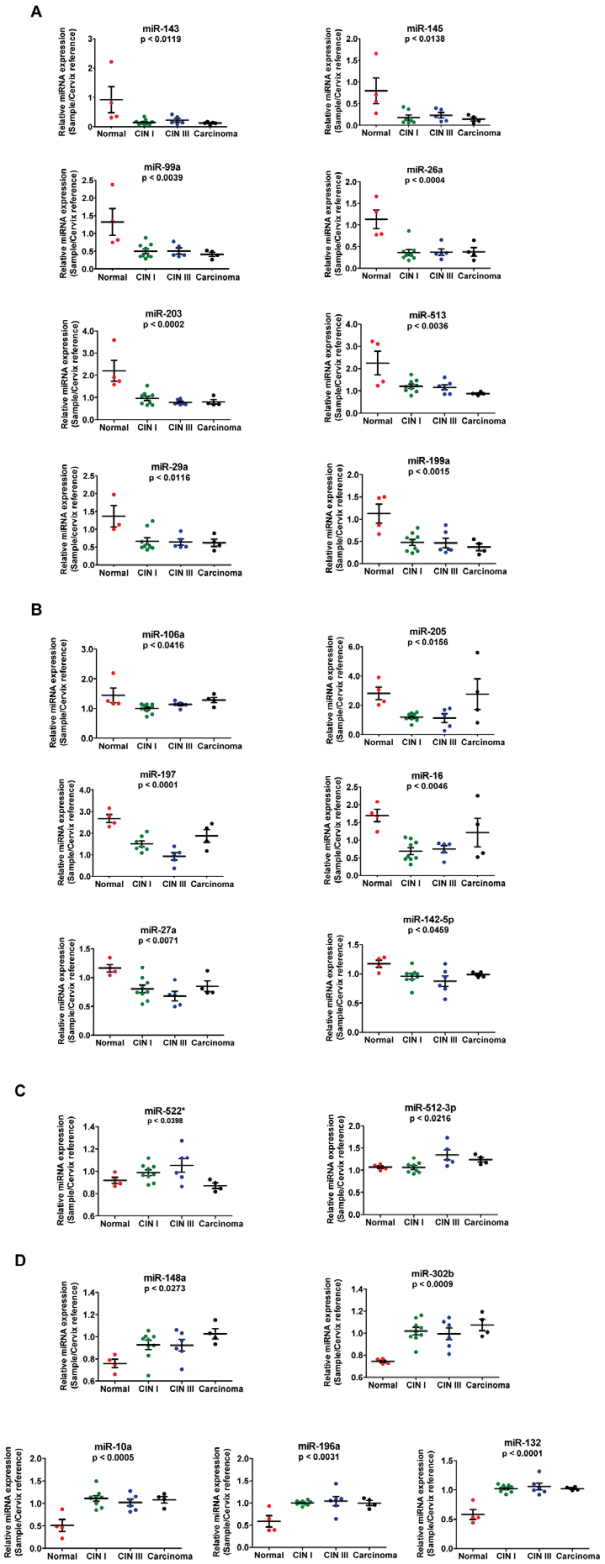
Scatter-plots representing the relative expression levels of 21 miRNAs. The relative expression of the 21 miRNAs that were deregulated are represented for normal cervix (red), CINI-cervical intraepithelial lesion grade I (green), CINIII-cervical intraepithelial lesion grade III (blue) and squamous cervical carcinoma (black) samples. **A**) 8 miRNAs that showed relative decrease in expression in the transition from normal cervix through premalignant dysplasia to cancer. **B**) 6 miRNAs that showed relative decreased expression in the transition from normal cervix to premalignant dysplasia, but returning to normal cervix expression levels in cancer. **C**) 2 miRNAs that showed relative increased expression in the transition from normal cervix to premalignant dysplasia, returing to normal cervix levels in cancer. **D**) 5 miRNAs showed relative increase in miRNA expression levels in the transition from normal cervix through premalignant dysplasia to cancer.

Using the methodology described above we were able to identify 21 miRNAs with statistically significant differential expression between the pool of normal samples (n = 4), 14 atypical dysplasia (CINI, n = 9 and CIN III, n = 5) and 4 cervical carcinoma (p<0.05). Eight miRNAs exhibited relative decreased expression with transition from normal cervix to atypical dysplasia to cancer (miR-26a, miR-143, miR-145, miR-99a, miR-203, miR-513, miR-29a, miR-199a) (Figure 4A). Six miRNAs displayed relative decreased expression in the transition from normal cervix to atypical dysplasia and increased expression in the transition from atypical dysplasia to cervical carcinoma, namely miR-106a, miR-205, miR-197, miR-16, miR-27a and miR-142-5p (Figure 4B). Two miRNAs exhibited relative increased expression in the transition from normal cervix to atypical dysplasia and decreased expression in the transition from atypical dysplasia to cervical carcinoma, namely miR-522* and miR-512-3p (Figure 4C). Five miRNAs displayed relative increased expression in the transition from normal cervix to atypical dysplasia to cancer, these were miR-148a, miR-302b, miR-10a, miR-196a and miR-132 (Figure 4 D).

Interestingly, several of these miRNAs are associated with fragile sites (FRAs) (Table S1). For example, miR-142-5p is located in FRA17B, while miR-196a and miR-29a are located in FRA12A and FRA7H, respectively. Furthermore, many of these miRNAs are located in chromosomal regions that are frequently deleted or amplified in several malignancies (Table S1). MicroRNA-143 and miR-145 are located in a region, which is deleted in prostate cancer, whereas miR-205 is located in the 12q14.1 region, which is amplified in lung cancer. Therefore, these data strongly suggest that miRNAs identified in this study are indeed relevant to cervical cancer.

### Predicted gene targets of miRNAs

MicroRNAs can regulate a large number of target genes and several databases based on various algorithms are available for predicting the targets of selected miRNAs. TargetScan 4.2, PicTar and miRanda were used to predict gene targets of 21 differentially expressed miRNAs with transition from normal to atypical dysplasia to cervical carcinoma (Table S2). The predictive targets were considered if they were predicted by at least two of the algorithms. To explore the biological significance of the predicted targets of the 21 deregulated miRNAs identified, we used Ingenuity Systems pathway analysis software (Table 2 and table S3). Reassuringly, the analysis identified “cancer” as the main disease associated with expression of those miRNAs. Cell death, cellular movement, cellular growth and proliferation and gene expression were the main biological processes related with these expression patterns. Regarding the canonical pathways, there was less consistency, with Wnt/β-catenin signaling found three times (miR-145, miR-199a and miR-132). ERK/MAPK signaling (miR-203), PTEN signaling (miR-27a), VEGF signalling (miR-10a), p53 signaling (miR-205) and apoptosis signalling (miR-512-3p) were found once (Table 2 and table S3). These data suggest that while the general pathways are related to cancer, the specific molecular pathways targeted by the deregulated miRNAs are variable and depend on each miRNA. This again points to high level of complexity of miRNA target selection and regulation.

**Table 2:** Ingenuity analysis of miRNA predictive target genes.

	Disease	Molecular and Cellular Function	Physiological System	Canonical Pathway
**miR-143**	Cancer	Cellular Growth and Proliferation	Connective Tissue Development and Function	PPAR Signaling
**miR-145**	Cancer	Cellular Movement	Tissue Development	IGF-1 Signaling
**miR-99a**	Cancer	Cell Death	Embryonic Development	Wnt/β-catenin Signaling
**miR-26a**	Cancer	Cellular Growth and Proliferation	Tissue Morphology	Synaptic Long Term Potentiation
**miR-203**	Cancer	Gene Expression	Nervous System Development and Function	ERK/MAPK Signaling
**miR-513-5p**	Cancer	Gene Expression	Hematological System Development and Function	Thrombopoietin Signaling
**miR-29a**	Cancer	Gene Expression	Tissue Development	Neurotrophin/TRK Signaling
**miR-199a-5p**	Cancer	Cell Death	Hematological System Development and Function	Wnt/β-catenin Signaling
**miR-106a**	Cancer	Gene Expression	Nervous System Development and Function	SAPK/JNK Signaling
**miR-205**	Cancer	Gene Expression	Tissue Morphology	p53 Signaling
**miR-197**	Cancer	Cellular Assembly and Organization	Connective Tissue Development and Function	GABA Receptor Signaling
**miR-16**	Cancer	Cell Cycle	Organismal Development	TGF-β Signaling
**miR-27a**	Cancer	Cellular Growth and Proliferation	Hematological System Development and Function	PTEN Signaling
**miR-142-5p**	Cancer	Gene Expression	Tissue Development	Regulation of Actin-based Motility by Rho
**miR-512-3p**	Genetic Disorder	Cell-to-Cell Signaling and Interaction	Nervous System Development and Function	Apoptosis Signaling
**miR-148a**	Cancer	Gene Expression	Nervous System Development and Function	Cell Cycle: G2/M DNA Damage Checkpoint Regulation
**miR-302b**	Cancer	Gene Expression	Tissue Development	Cell Cycle: G1/S Checkpoint Regulation
**miR-10a**	Cancer	Gene Expression	Organismal Development	VEGF Signaling
**miR-196a**	Cancer	Cellular Growth and Proliferation	Connective Tissue Development and Function	Chemokine Signaling
**miR-132**	Cancer	Gene Expression	Nervous System Development and Function	Wnt/β-catenin Signaling

The top pathway is indicated for each parameter.

Finally, we have identified a number of down-regulated miRNAs whose targets are up-regulated in cancer, including the oncogenic protein KRAS and MYCN, mitogen-activated protein (MAP) kinases and the anti-apoptotic proteins BCL2, BCL2L2 and MCL-1 among others (see table S2). These data provide further support for a pivotal role of the miRNAs identified in this study in cervical cancer.

## Discussion

Previous studies support the hypothesis that specific miRNA expression signatures in various types of human cancers can be associated with diagnosis, prognosis and response to chemotherapy [22]. Since specific miRNAs may have crucial roles in cancer pathogenesis and progression through their effects on various molecular pathways, a better understanding of miRNA expression in human cancer may reveal novel molecular pathways or novel mechanisms of activation of known pathways. Interestingly, several miRNAs are frequently located in cancer-related genomic regions, which include minimal regions of amplification, loss of heterozygosity, fragile sites, common breakpoint regions in or near oncogenes or tumour suppressor genes and at or near HPV integration sites [14].

This study demonstrated that miRNAs are aberrantly expressed in human cervical cancer and cervical pre-neoplasic lesions. The overall miRNA expression profile could not clearly separate normal, pre-neoplasic and cancer tissues probably due to the high variability among normal cervical samples. One reason for this variability could be the presence of the HPV in normal samples since these samples were collected in normal epithelium adjacent to the lesion and HPV detection by PCR showed both HPV presence and absence in normal samples (data not shown). Martinez and co-workers showed that HPV type 16 and 18 can alter miRNA expression in cases where the virus is both integrated and epissomal [17]. This could explain the variability observed in normal cervical tissues since the commercial RNA from normal cervix used as common reference was HPV negative. The confirmation of the differential expression pattern obtained by microarray analysis of five miRNAs by Taqman qRT-PCR excluded the possibility that the high variability among samples was technical. MicroRNA expression measurement in HeLa cells by Taqman qRT-PCR compared with cervical carcinoma tissues showed a similar tendency of miRNA expression, with exception of miR-10a, although the values of fold induction were not the same. This could be due to biological differences between the immortalized cell line and fresh biopsies. By pooling normal cervical samples to reduce the variability observed we were able to separate normal, pre-neoplasic and cancer samples and identified a number of miRNAs whose expression was altered in human cervical cancer and pre-neoplasic samples. Therefore, a degree of caution is required when carrying out miRNA profiling using human biopsies. Indeed, natural genetic variation in the human population, latent viral infections, aging and health problems may be important sources of biological variation in the expression profiles. Such problems may explain the inconsistencies in miRNA profiles described in the literature (Table 3) and may contribute to erroneous interpretations of the data and affect future therapeutic strategies based on RNAi.

**Table 3:** Comparison of miRNA expression profiles in cervical cancer studies published in the literature and the present study.

	Lui *et al.* [15]	Martinez *et al.* [17]	Lee *et al.* [18]	Wang *et al.* [20]		Pereira *et al.*
	Cervical cell lines	Cervical cell lines	Cervical tissues	Cervical tissues	Cervical cell lines	Cervical tissues
**miR-143**	down	down	up	down		down
**miR-145**		down	up	down		down
**miR-21**	up		up		up	
**miR-199a**			up	down		down
**miR-203**		up	down			down
**miR-155**			up		up	
**miR-29a**			up	down		down
**miR-146a**			up	down	up	
**miR-218**		down	up	down	down	
**miR-148a**					up	up
**miR-10a**						up
**miR-196a**						up

The table only highlights miRNAs whose expression showed variability between the studies published in literature.

### miRNAs down-regulated between normal cervix and pre- and neoplasic samples

At least eight miRNAs showed significant down-regulation between normal cervical samples and the pre-neoplasic and neoplasic samples, namely miR-143, miR-145, miR-99a, miR-26a, miR-203, miR-513, miR-29a and miR-199a. The abundance of miR-143 and miR-145 was sharply reduced between the normal cervical samples and cervical pre-neoplasic and cancer tissues, which is in agreement with previous cervical cancer studies [15], [20]. Similarly, expression of miR-143 and miR-145 was reduced in different tumour types, e.g., colorectal tumours [23], breast, prostate and B cell lymphoma [24], [25], suggesting that those miRNAs may have a suppressor role in a wide range of tumours. Previous studies also showed that miR-143 and miR-145, which are expressed from the same precursor [26], showed reduced expression in HPV-induced pre-neoplasic lesions suggesting that they might be involved in cervical carcinogenesis. These miRNAs are located in sites of frequent chromosomal instability [14] resulting in loss- or gain-of-function of their activity, which seems to be a key event in the genesis of a variety of cancers. Among the down-regulated miRNAs was miR-199a, which is also down-regulated in hepatocellular carcinoma [27] and ovarian cancer [28]. This contradicts data from a recent cervical cancer study, which showed that miR-199a was up-regulated in early stage invasive squamous cell carcinomas [18]. The same study showed that an anti-miR-199a inhibits cell growth, suggesting that miR-199a can promote cell proliferation. These discrepancies may be technical since we used home-made arrays for the identification of miRNA expression while Lee and co-workers used the TaqMan miRNA assay [18]. Also, the samples that we have used as normal control were different from those used by Lee and colleagues and the control sample could also have influenced the outcome of the miRNA profiling analysis [29]. Similar discrepancies were also observed for miR-26a and miR-29a, which were down-regulated in our study and up-regulated in the study of Lee and co-workers [18] (Figure 3). Further source of miRNA expression heterogeneity in cervical cancer is related to the cellular levels of Drosha, at least in cervical squamous cell carcinoma [16]. We have not quantified Drosha levels by western blot analysis, but this may be an important variable to consider in future studies.

The microarray data of miR-26a showed that its expression was decreased in pre-neoplasic and cancer in comparison with normal cervical samples. Interestingly, miR-26a-1 is located in sites of frequent chromosomal instability [14] and it is down-regulated in many other tumour types, namely in thyroid anaplastic [30] and breast carcinomas [31]. miR-29a is located in the fragile site FRA7H, is deleted in prostate cancer [32] and is down-regulated in serous ovarian cancer [33]. We also have found that miR-29a can potentially target BCL2L2, VEGFA and CDK6, which are involved in cancer initiation and progression (see table S2). Therefore, these miRNAs may be involved in the cervical carcinogenesis mechanism. Other down-regulated miRNAs included miR-99a, which is deleted in the lung cancer cell line MA17 [34] and in serous ovarian cancer [33] and miR-203 whose gene is deleted in nasopharyngeal carcinoma [35] and is down-regulated in cervical cancer [18]. Finally, miR-513 which targets the oncogene *KRAS*, the c-myc binding protein (MYCBP), MAPK7 which is a member of the mitogen-activated signal transduction pathway and the CD44 protein, which is a cell-surface glycoprotein involved in cell-cell interactions, cell adhesion and migration, was also down-regulated in our data set (Figure 3). A recent study showed that miR-200a could predict patient survival in cervical cancer and functional studies suggest that it may affect the metastatic potential of cervical cancer cells [21]. This miRNA is also down-regulated between our normal cervical samples and the pre-neoplasic and neoplasic samples (p<0.012; data not shown), suggesting that some of those miRNAs that we found down-regulated from normal to cancer samples could be use as prognostic markers.

### miRNAs deregulated between normal cervix and pre-neoplasic samples

Among the miRNAs that were down-regulated between normal and pre-neoplasic cervical samples, but had increased expression in cervical cancer samples, were miR-106a, miR-205, miR-197, miR-16, miR-27a and miR-142-5p. Down-regulation of these miRNAs in CIN I and CIN III samples suggested that they may play an important role in cervical cell abnormal transformation by HPV infection, but are not directly involved in progression to malignant state since their expression is nearly restored to the levels of normal cervical samples. Interestingly, miR-142-5p is located in 17q23, which is in proximity to the t(8, 17) breakpoint in B cell acute leukemia and also within the minimal amplicon in breast cancer and near the FRA17B site, a target for HPV16 integration in cervical tumours [14]. We were able to predict several important targets of miR-106a using miRanda, PicTar and TargetScan, namely the microtubule-associated protein MAP7, which is predominantly expressed in cells of epithelial origin and is essential for cell polarization and differentiation, the early growth response protein EGR2, the BCL2L2 protein, which is an anti-apoptotic member of the BCL2 protein family, the mitogen-activated protein kinase MAPK9, the oncogene *MYCN*, as well as the tumour suppressor gene *TP53INP*. This may suggest that miR-106a may play an important role during the initial stages of atypical growth by targeting proteins involved in cell growth and proliferation rather than being directly involved in cervical carcinogenesis, depending on its cellular concentration. Our data set suggests that miR-16 and miR-205 may have an oncogenic role or at least they seem to promote abnormal cell growth in basal epithelial cells since they are down-regulated in CINI and CINIII compared with normal cervical tissue and are up-regulated in cervical samples. In addition, they share putative targets, which are involved in cell growth, migration and proliferation, namely the vascular endothelial growth factor A (VEGFA) and the anti-apoptotic protein BCL2. Also, miR-16 is deleted or down-regulated in Chronic Lymphocytic Leukemia and miR-205 is located in a chromosomal region, which is amplified in lung cancer and is down-regulated in human prostate cancer. The miR-27a and miR-197 showed expression patterns that go from down-regulation between normal cervical tissues and CINI and CINIII samples to slight up-regulation in cervical cancer. In breast cancer cells miR-27a acts as an oncogene by targeting *Myt-1*, which blocks cell cycle progression at G_2_-M and through regulation of Sp proteins that have an important role in angiogenesis and growth of cancer cells [36]. Our study suggests that miR-27a acts as an oncogene, particularly in the early stages of cervical cell abnormal transformation, because it targets the EGFR protein, the cyclin-dependent kinases CD28 and CD44, the oncogenes *KRAS* and *MYCN*, the MAPK7, the VEGFB and VGF. The expression pattern of miR-197 is similar to that of miR-27a suggesting a similar role. The miR-512-3p and miR-522* were up-regulated between the normal cervical tissues and CINI and CINIII samples and had normal expression in cervical carcinoma. The expression pattern of miR-512-3p and its putative targets (Table S2) suggest that this miRNA may play an important role in the development of dysplasia in cervical tissues and is apparently less important during cell invasion. Interestingly, the predictive targets of miR-152-p includes mitogen-activated protein kinase kinases MAP3K11 and MAP2K4, which activate the mitogen-activated protein kinase pathway implicated in the development and progression of many human cancers and the oncogene *erbb4* and the cell adhesion protein 4 CADM4 involved in cell-cell adhesion. And also the autophagy TP53INP1 and TP53INP2 proteins [37]. Thus, depending on the cell state and miRNA level, miR-521-3p may function as an oncogene or tumour suppressor and it may play a role in cervical dysplasia. For miR-522* we could not find any predictive targets, but the miRNA expression pattern suggests that this miRNA may also play a role in cervical abnormal transformation.

### miRNAs up-regulated in pre- and neoplasic samples

Several miRNAs, namely miR-148a, miR-302b, miR-10a, miR-196a and miR-132 were up-regulated in CINI samples. This increase in expression was maintained in CINIII and cervical carcinoma. The up-regulation of miR-10a and miR-132 confirmed the data from a previous cervical carcinoma study where these miRNAs were also up-regulated [18]. Interestingly, miR-10a and miR-196a target homeobox (HOX) genes, which are a family of transcription factors that control developmental processes [38]. Up-regulation of these transcriptional activators was initially thought to enhance oncogenesis, however, both loss and gain of *HOX* gene expression are related to carcinogenesis [39]. The deregulation of *HOX* genes is associated with both leukemia and solid malignancies [40]–[43], suggesting that those two miRNAs contribute to cervical abnormal cell transformation and progression of cervical carcinoma through deregulation of the *HOX* genes. Finally, miR-196a is located in the rare folate sensitive fragile site FRA12A, the other two miRNAs that belong to this group are miR-148a and miR-302b. These miRNAs target the tumour suppressor genes, *PTEN*, *TP53INP1* and *TP53INP2*, and other proteins involved in cancer development and progression, such as BCL2L2, ERBB4, MAPK9, MCL1, MYCN, VEGFA and VEGFR1.

In conclusion, our study showed that natural miRNA expression variability among cervical samples may complicate the use of miRNA profiling in clinical diagnostics. The data provides an explanation for the low consistency of cervical cancer miRNA profiles available in the literature. We were able to minimize this biological problem and were able to identify miRNAs whose expression was significantly deregulated. Our strategy to use multiple miRNA target prediction algorithms allowed us to compile the largest list of putative targets of the miRNAs that are differently expressed in cervical cancer. Genes involved in cancer, cell death, cellular movement, cellular growth and proliferation and gene expression processes were identified, which to certain extent, validated our miRNA profiling data. Indeed, several *HOX* genes were identified, which is in agreement with data obtained in previous cervical carcinoma studies. In addition, the well known oncogenes, *MYCN*, *KRAS* and *VEGF*s and the tumour suppressors genes *TP53INP* and *PTEN* were identified as targets of specific miRNAs. Therefore, our results increase the understanding of the molecular basis of human cervical cancer and confirm that aberrant expression of miRNA genes may be important for the pathogenesis of this human neoplasia by controlling or fine-tunning gene expression. The putative miRNA targets provide new tools to better characterize cervical carcinogenesis. In conclusion, our data supports a role for miRNAs in cervical cancer and suggest that large scale miRNA profiling studies should be carried out in order to obtain a robust miRNA signature for this disease.

## Materials and Methods

### Ethics statement

At the time of initial diagnosis, all patients had provided consent in the sense that their tumour samples could be used for investigational purposes. Institutional approval from local research ethical committee was obtained for the conduct of the study (Comissão de Ética dos HUC - Hospitais da Universidade de Coimbra). Data were analyzed anonymously. Patients provided written consent so that their samples and clinical data could be used for investigational purposes.

### Tissue cervical samples

All tissue specimens were used with Hospital of the University of Coimbra Ethical Committee approval and informed written consent was obtained from patients. Each sample used in this study was from a different patient of the Gynaecology Department of the Hospital of the University of Coimbra. The specimens used were: i) 4 pre-treatment squamous cell cervical carcinoma. ii) Lesional epithelium frozen biopsies of 7 cases of high-grade SIL (CINII, n = 2 and CIN III, n = 5) and 9 cases of low-grade SIL (CIN I). In all cases the biopsy was composed of abnormal epithelium as confirmed by cytology and histology. iii) 19 samples were from normal cervix. Extracted tissue specimens were immediately snap-frozen in liquid nitrogen and stored at −80°C until the preparation of total RNA. The 19 normal cervix RNA samples were pooled to obtain the normal cervix pooled sample.

### miRNA microarray production

The miRNA microarrays used in this study were printed by the National DNA-Microarray Facility located at the University of Aveiro, Portugal. The arrays contained a total of 381 probes spotted in quadruplicate. The 381 probes represent 281 known human miRNAs, 49 mouse miRNAs, 14 rat miRNAs, 33 predicted human miRNAs and 4 control probes (Ambion). In addition, we designed four oligonucleotides (∼100 bp) containing no homology to any known RNA sequence and generated their corresponding synthetic RNAs by *in vitro* transcription using the Ambion MEGAscript® T7 Kit. Various amounts of these synthetic RNAs were added to the human miRNA samples before analysis, external controls.

Oligonucleotides probes were dissolved in 3XSSC buffer at a concentration of 20 µM. and printed onto Nexterion E slides (SCHOTT) using a MicroGrid II compact spotter. Printed slides were further processed according to manufacture's recommendations.

### RNA extraction and quantitation

Total RNA was extracted using the miRVana™ miRNA isolation kit (Ambion) according to the manufacture's instructions. RNA quantity and quality was assessed using the Nanodrop and Agilent 2100 bioanalyzer systems, respectively. Samples with a RIN number above 7 were used in the study.

### miRNA microarray hybridization

Total RNA from test and reference (First Choice Human Cervix RNA, Ambion) samples were labelled using the miRacULS II miRNA labelling kit (Kreatech) according to the manufacture's instructions. Briefly, 3 µg of total RNA from test and reference samples were incubated with Cy5- and Cy3-ULS, respectively for 15 min at 85°C. The labelled RNAs were purified to remove non-reacted Cy-ULS followed by isolation of the labelled small RNA fraction, to produce a fluorescently-labelled small RNA sample for microarray analysis. Dye incorporation was monitored by UV-visible spectroscopy. Hybridization was carried out in the home made miRNA microarray at 42°C for 16h. Slides were washed following the manufacture's recommendations and immediately scanned using an Agilent G2565AA microarray scanner.

### Computational analysis of miRNA microarray data

Microarray images were analyzed using Quantarray v3.0 software (PerkinElmer). Cy3 and Cy5 median pixel intensity values were background subtracted, normalized and subject to further analysis. Data points were removed when intensity values for both dyes were below 200% of background and absent calls were removed before subsequent statistical analysis. A global median normalization of cervical microarray data was applied using BRB-ArrayTools v3.4.0 software. Cy5/Cy3 ratios were obtained and log-transformed (base 2). Differentially expressed miRNAs from each sample type were identified using the Significance Analysis of Microarrays (SAM) software. One-way ANOVA, Tukey's Multiple Comparison Test, was performed to identify miRNAs that demonstrated statistically significant differentially expression between, normal cervix, CIN I, CIN III and cervical squamous cell carcinoma.

### Microarray Data Submission

Microarray data submission for human arrays is MIAME compliant. The raw data has been submitted in Gene Expression Omnibus (GEO) database and has been given the following accession numbers: GSE19611; GPL7534; GSM489127–GSM489169.

### Taqman miRNA quantitative real-time Polymerase Chain Reaction Analysis

Quantification of microRNA expression was carried out using TaqMan MicroRNA Assay kits according to manufacturer's protocol (Applied Biosystems, Foster City, CA, USA). Prefabricated TaqMan MicroRNA Assays were applied for the investigation of hsa-miR-143, hsa-miR-197, has-miR-27a, has-miR-10a and has-miR-148a. We also quantified transcripts of U6 small RNA (RNU6B) as an endogenous control for normalizing the levels of target miRNA. RNU6B is a widely used endogenous reference RNA in miRNA quantification experiment because RNU6B is not regulated under the experimental conditions and shows a constant level of expression and similar abundance to the target miRNA. CDNA was generated using the Taqman MicroRNA Reverse Transcription (RT) Kit according to the manufacturer's instructions. Reverse transcriptase reactions contained 10 ng of total RNA as the template, 3 µL 5× RT primer, 1.5 µL 10× RT buffer, 0.15 µL 100 mM dNTPs, 1 µL MultiScribe reverse transcriptase, 0.19 µL RNase Inhibitor, and 4.16 µL nuclease – free water. The 15 µL reactions were mixed and incubated for 30 minutes at 16°C, 30 minutes at 42°C, 5 minutes at 85°C, and then held at 4°C. All reverse transcriptions and no-template controls were run at the same time following the RT step. Real-time Quantitative PCR was carried out using the ABI Prism 7500 Sequence Detector System (Applied Biosystems). The 20-µL PCR reactions contained 1.33 µL RT product, 10 µL TaqMan® Universal PCR Master Mix, No AmpErase® UNG, 7.67 µl nuclease – free water and 1 µL of 20× MicroRNA Assay. Reactions were incubated in a 96-well optical plate and the cycling began with template denaturation and hot start Taq activation at 95°C for 10 minutes, followed by 40 cycles of 95°C for 15 seconds and 60°C for one minute. The threshold cycle data (CT) and baselines were determined using auto settings. All assays including no template controls were done in triplicate. Relative quantification of miRNA expression was calculated by the 2^−ΔΔCT^ method [44], where control sample was the normal cervix reference (Ambion).

## Supporting Information

Table S1 Twenty-one miRNAs differentially expressed in clinical cervical samples.
(0.07 MB DOC)

Table S2 Predictive gene targets of 21 miRNAs differentially expressed in clinical cervical samples.
(0.37 MB XLS)

Table S3 Ingenuity analysis of miRNA predictive target genes. The top three pathways are indicated for each parameter. Pathways found more that 3 times are indicated in bold.
(0.10 MB DOC)
