# Supplementary material for: MicroRNA Expression Variability in Human Cervical Tissues
Source: PLoS One. 2010 Jul 26;5(7):e11780. doi: 10.1371/journal.pone.0011780 (PMC2909898; doi:10.1371/journal.pone.0011780)
Supplement: Table S1 — Twenty-one miRNAs differentially expressed in clinical cervical samples. (0.07 MB DOC) [file pone.0011780.s001.doc]

| **miRNA** | **Location^a^** | **FRA association^b^** | **Cancer-associated genomic regions^b^** | **Deregulation in other cancers** |
| --- | --- | --- | --- | --- |
| miR-143 | 5q33.1 |  | Del-prostate cancer | Down-regulated in breast cancer and lung cancer [43] and deleted in prostate cancer |
| miR-145 | 5q33.1 |  | Del-prostate cancer | Down-regulated in breast cancer and lung cancer [43] and deleted in prostate cancer |
| miR-99a | 21q21.1 |  | Del-Lung Cancer (cell line MA17) | Down-regulated in Squamous Cell Carcinoma of Tongue [44] |
| miR-26a | 3p22.2, 12q14.1 |  |  |  |
| miR-203 | 14q32.33 |  | Del-nasopharyngeal carcinoma |  |
| miR-513-5p | Xq27.3 |  |  |  |
| miR-29a | 7q32.3 | FRA7H | Del-prostate cancer | Down-regulated in lung cancer [43] |
| miR-199a-5p | 19p13.2, 1q24.3 |  |  | Up-regulated in lung, prostate and pancreas cancer [30] |
| miR-106a |  |  |  | Up-regulated in colon, pancreas and prostate cancer [30] |
| miR-205 | 1q32.2 |  | Amp-lung cancer |  |
| miR-197 | 1p13.3 |  |  |  |
| miR-16 | 13q14.3, 3q26.1 |  | Del-B-Chronic Lymphocytic Leukemia (B-CLL); lipoma; CLL; adult lymphoblastic Leukemia; head-and-neck-Squamous cell carcinoma; oral cancer |  |
| miR-27a | 19p13.2 |  |  |  |
| miR-142-5p | 17q22 | FRA17B | Amp-Breast cancer  t(8;17)-Prolymphocytic leukemia |  |
| miR-522* | 19q13.41 |  |  |  |
| miR-512-3p | 19q13.41 |  |  |  |
| miR-148a | 7p15.2 |  |  |  |
| miR-302b | 4q25 |  |  |  |
| miR-10a | 17q21.32 |  |  |  |
| miR-196a | 17q21.32, 12q13.13 | FRA12A |  |  |
| miR-132 | 17p13.3 |  |  | Up-regulated in Squamous Cell Carcinoma of Tongue [44] |
| Amp: amplification; Del: Deletion; t: translocation  ^a^ Information was obtained from miRBase  ^b^ Information was obtained from previous report [14] | | | |  |

**Table S1**
