# Supplementary material for: MicroRNA Expression Variability in Human Cervical Tissues
Source: PLoS One. 2010 Jul 26;5(7):e11780. doi: 10.1371/journal.pone.0011780 (PMC2909898; doi:10.1371/journal.pone.0011780)
Supplement: Table S3 — Ingenuity analysis of miRNA predictive target genes. The top three pathways are indicated for each parameter. Pathways found more that 3 times are indicated in bold. (0.10 MB DOC) [file pone.0011780.s003.doc]

**Table S3**

|  | **miR-143** | **miR-145** | **miR-99a** | **miR-26a** | **miR-203** | **miR-513-5p** | **miR-29a** | **miR-199a-5p** | **miR-106a** | **miR-205** |
| --- | --- | --- | --- | --- | --- | --- | --- | --- | --- | --- |
| Disease | **Cancer** | **Cancer** | **Cancer** | **Cancer** | **Cancer** | **Cancer** | **Cancer** | **Cancer** | **Cancer** | **Cancer** |
|  | **Neurological Disease** | **Neurological Disease** | **Reproductive System Disease** | **Reproductive System Disease** | **Neurological Disease** | **Genetic Disorder** | Dermatological Diseases and Conditions | **Neurological Disease** | **Reproductive System Disease** | **Genetic Disorder** |
|  | Gastrointestinal Disease | **Reproductive System Disease** | **Genetic Disorder** | **Connective Tissue Disorders** | **Genetic Disorder** | **Neurological Disease** | **Genetic Disorder** | **Reproductive System Disease** | **Genetic Disorder** | Respiratory Disease |
| Molecular and Cellular Function | **Cellular Growth and Proliferation** | **Cellular Movement** | **Cell Death** | **Cellular Growth and Proliferation** | **Gene Expression** | **Gene Expression** | **Gene Expression** | **Cell Death** | **Gene Expression** | **Gene Expression** |
|  | **Cell Death** | **Cellular Development** | **Cellular Growth and Proliferation** | **Cell Death** | **Cell Death** | **Cellular Growth and Proliferation** | **Cellular Growth and Proliferation** | **Cellular Development** | **Cell Death** | **Cell Cycle** |
|  | **Cell Morphology** | **Cellular Growth and Proliferation** | **Cellular Development** | **Cell Morphology** | **Cell Cycle** | **Cellular Development** | **Cell-To-Cell Signaling and Interaction** | Cell Signaling | **Cell Cycle** | **Cellular Growth and Proliferation** |
| Physiological System | **Connective Tissue Development and Functio** | **Tissue Development** | **Embryonic Development** | **Tissue Morphology** | **Nervous System Development and Function** | **Hematological System Development and Function** | **Tissue Development** | **Hematological System Development and Function** | **Tissue Morphology** | **Cardiovascular System Development and Function** |
|  | **Hematological System Development and Function** | **Connective Tissue Development and Function** | **Organismal Development** | **Embryonic Development** | **Cardiovascular System Development and Function** | **Hematopoiesis** | **Nervous System Development and Function** | **Hematopoiesis** | **Cardiovascular System Development and Function** | **Tissue Morphology** |
|  | **Tissue Morphology** | **Hematological System Development and Function** | **Skeletal and Muscular System Development and Function** | **Nervous System Development and Function** | Hair and Skin Development and Function | **Tumour Morphology** | **Skeletal and Muscular System Development and Function** | **Connective Tissue Development and Function** | **Connective Tissue Development and Function** | **Connective Tissue Development and Function** |
| Canonical Pathways | PPAR Signaling | IGF-1 Signaling | **Wnt/β-catenin Signaling** | Synaptic Long Term Potentiation | ERK/MAPK Signaling | Thrombopoietin Signaling | Neurotrophin /TRK Signaling | **Wnt/β-catenin Signaling** | SAPK/JNK Signaling | p53 Signaling |
|  | Neuregulin Signaling | **TGF-β Signaling** | Chondroitin Sulfate Biosynthesis | Clathrin-mediated Endocytosis | **Synaptic Long Term Potentiation** | PPAR Signaling | TR/RXR Activation | N-Glycan Biosynthesis | Cell Cycle: G1/S Checkpoint Regulation | Hypoxia Signaling in the Cardiovascular System |
|  | **Synaptic Long Term Potentiation** | Fcγ Receptor-mediated Phagocytosis in Macrophages and Monocytes | Keratan Sulfate Biosynthesis | Inositol Phosphate Metabolism | PDGF Signaling | **PTEN Signaling** | B Cell Receptor Signaling | Glycosphingolipid Biosynthesis - Neolactoseries | Role of BRCA1 in DNA Damage Response | **TGF-β Signaling** |

|  | **miR-197** | **miR-16** | **miR-27a** | **miR-142-5p** | **miR-512-3p** | **miR-148a** | **miR-302b** | **miR-10a** | **miR-196a** | **miR-132** |
| --- | --- | --- | --- | --- | --- | --- | --- | --- | --- | --- |
| Disease | **Cancer** | **Cancer** | **Cancer** | **Cancer** | **Genetic Disorder** | **Cancer** | **Cancer** | **Cancer** | **Cancer** | **Cancer** |
|  | **Reproductive System Disease** | **Neurological Disease** | **Neurological Disease** | **Genetic Disorder** | **Neurological Disease** | **Reproductive System Disease** | **Reproductive System Disease** | **Reproductive System Disease** | **Genetic Disorder** | **Reproductive System Disease** |
|  | **Neurological Disease** | Developmental Disorder | **Genetic Disorder** | **Neurological Disease** | **Cancer** | Infection Mechanism | **Genetic Disorder** | **Connective Tissue Disorders** | Dermatological Diseases and Conditions | **Connective Tissue Disorders** |
| Molecular and Cellular Function | Cellular Assembly and Organization | **Cell Cycle** | **Cellular Growth and Proliferation** | **Gene Expression** | **Cell-to-Cell Signaling and Interaction** | **Gene Expression** | **Gene Expression** | **Gene Expression** | **Cellular Growth and Proliferation** | **Gene Expression** |
|  | **Cell Morphology** | **Gene Expression** | **Gene Expression** | **Cellular Growth and Proliferation** | **Cell Death** | **Cellular Development** | **Cellular Growth and Proliferation** | **Cell Cycle** | **Gene Expression** | **Cell Morphology** |
|  | **Cell-to-Cell Signaling and Interaction** | **Cell Death** | **Cell Cycle** | **Cellular Movement** | **Cellular Development** | **Cellular Movement** | **Cell Death** | **Cellular Development** | **Cell Cycle** | **Cellular Growth and Proliferation** |
| Physiological System | **Connective Tissue Development and Function** | **Organismal Development** | **Hematological System Development and Function** | **Tissue Development** | **Nervous System Development and Function** | **Nervous System Development and Function** | **Tissue Development** | **Organismal Development** | **Connective Tissue Development and Function** | **Nervous System Development and Function** |
|  | **Nervous System Development and Function** | **Embryonic Development** | **Organismal Development** | **Nervous System Development and Function** | **Tissue Development** | **Embryonic Development** | **Nervous System Development and Function** | **Embryonic Development** | **Organismal Development** | **Reproductive System Development and Function** |
|  | Endocrine System Development and Function | **Tissue Development** | **Hematopoiesis** | **Skeletal and Muscular System Development and Function** | **Embryonic Development** | **Tissue Development** | **Organismal Development** | **Cardiovascular System Development and Function** | **Hematological System Development and Function** | **Skeletal and Muscular System Development and Function** |
| Canonical Pathway | GABA Receptor Signaling | **TGF-β Signaling** | **PTEN Signaling** | Regulation of Actin-based Motility by Rho | Apoptosis Signaling | Cell Cycle: G2/M DNA Damage Checkpoint Regulation | Cell Cycle: G1/S Checkpoint Regulation | VEGF Signaling | Chemokine Signaling | **Wnt/β-catenin Signaling** |
|  | Glutamate Receptor Signaling | **PTEN Signaling** | B Cell Receptor Signaling | Chemokine Signaling | Death Receptor Signaling | Neuregulin Signaling | Hypoxia Signaling in the Cardiovascular System | Caveolar-mediated Endocytosis | α-Adrenergic Signaling | IGF-1 Signaling |
|  | **Synaptic Long Term Potentiation** | Axonal Guidance Signaling | PPARα/RXRα Activation | Clathrin-mediated Endocytosis | Lysine Degradation | **TGF-β Signaling** | Cell Cycle: G2/M DNA Damage Checkpoint Regulation | Estrogen Receptor Signaling | BMP signaling pathway | ERK/MAPK Signaling |
